# P-1838. Improving Hepatitis C Virus Screening for Adults in a Safety-net Primary Care Clinic

**DOI:** 10.1093/ofid/ofaf695.2007

**Published:** 2026-01-11

**Authors:** Lwin Mya, Aparna Rathnam, TuTu Mon, Marlon E Brewer

**Affiliations:** Elmhurst Hospital, Rego Park, NY; Icahn School of Medicine at Mount Sinai, Queens, New York; Elmhurst Hospital, Rego Park, NY; Elmhurst Hospital, Rego Park, NY

## Abstract

**Background:**

The Centers for Disease Control and Prevention (CDC) recommends universal screening for hepatitis C among all adults aged 18 to 79 irrespective of risk factors. Given the absence of a preventive vaccine, early diagnosis is the best way to prevent complications such as liver failure and cirrhosis. The goal of our quality improvement project was to increase the percentage of hepatitis C screening in our primary care clinic patients aged 18 to 79 from a baseline of 74% to 80% between May 2024 and May 2025.

**Figure:**
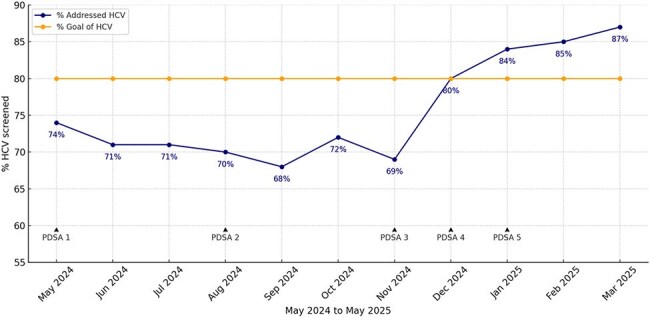
Hepatitis C Screening Percentage By Month Improving Hepatitis C Virus Screening for Adults in a Safety-net Primary Care Clinic

**Methods:**

The presence of hepatitis C screening serology in all eligible individuals presenting to the primary care clinic at our urban safety-net hospital was evaluated monthly through electronic chart reviews. For PDSA cycle 1, in May 2024, a red-text Smartphrase for hepatitis C screening was added to the ambulatory clinic note template, prompting providers to review and fill out hepatitis C screening status before signing the note. PDSA cycle 2, in August 2024, involved educating residents about hepatitis C screening. PDSA cycle 3 started in November 2024 and involved sending monthly reminder emails to providers who missed hepatitis C screening for eligible patients. During PDSA cycle 4, in December 2024, provider education was implemented during monthly ambulatory care meetings. The importance of hepatitis C screening regardless of risk factors was emphasized. PDSA cycle 5, in January 2025, focused on patients’ awareness: patients were sent reminders about their unscreened hepatitis C status via MyChart application.

**Results:**

After PDSA 1, the percentage of eligible patients screened for Hepatitis C decreased from 74% to 70%, and it further decreased to 68% after PDSA 2. This result is not entirely surprising since education alone in Quality Improvement is known to be at best modestly effective and temporary. After PDSA 3, the screened percentage increased to 80% and further increased to 84% after PDSA 4 and 87% after PDSA 5.

**Conclusion:**

In our project, outcomes improved following the reinforcement of hepatitis C screening guidelines during monthly meetings. Prior to this project, providers were screening only high-risk individuals, but after providing education on the updated guidelines and implementing monthly email notifications, we exceeded our goal.

**Disclosures:**

All Authors: No reported disclosures

